# Wnt-11 as a Potential Prognostic Biomarker and Therapeutic Target in Colorectal Cancer

**DOI:** 10.3390/cancers11070908

**Published:** 2019-06-28

**Authors:** Irantzu Gorroño-Etxebarria, Urko Aguirre, Saray Sanchez, Nerea González, Antonio Escobar, Ignacio Zabalza, José Maria Quintana, Maria dM Vivanco, Jonathan Waxman, Robert M. Kypta

**Affiliations:** 1 Cancer Heterogeneity Lab, CIC bioGUNE, 48160 Derio, Spain; 2 Research Unit, Galdakao-Usansolo Hospital, 48960 Galdakao, Spain; 3 Kronikgune Institute, Health Services Research on Chronic Patients Network (REDISSEC), 48902 Bilbao, Spain; 4 Research Unit, Basurto University Hospital, Osakidetza, 48013 Bilbao, Spain; 5 Department of Pathology, Galdakao-Usansolo Hospital and Biocruces-Bizkaia Institute, 48960 Galdakao, Spain; 6 Department of Surgery and Cancer, Imperial College London, W12 0NN London, UK

**Keywords:** Wnt signaling, Wnt-11, frizzled, colorectal cancer

## Abstract

The expression of the secreted factor Wnt-11 is elevated in several types of cancer, including colorectal cancer, where it promotes cancer cell migration and invasion. Analysis of colorectal cancer gene expression databases associated WNT11 mRNA expression with increased likelihood of metastasis in a subset of patients. WNT11 expression was correlated with the expression of the Wnt receptors FZD6, RYK, and PTK7, and the combined expression of WNT11, FZD6 and RYK or PTK7 was associated with an increased risk of 5-year mortality rates. Immunohistochemical analysis of Wnt-11 in a cohort of 357 colorectal cancer patients found significantly higher Wnt-11 levels in tumors, compared with benign tissue. Elevated Wnt-11 levels occurred more frequently in rectal tumors than in colonic tumors and in tumors from women than men. In univariate analysis, increased Wnt-11 expression was also associated with tumor invasion and increased 5-year mortality. High Wnt-11 levels were not associated with high levels of nuclear β-catenin, suggesting Wnt-11 is not simply an indicator for activation of β-catenin-dependent signaling. Expression of Wnt-11 in colorectal cancer cell lines expressing low endogenous Wnt-11 inhibited β-catenin/Tcf activity and increased ATF2-dependent transcriptional activity. WNT11 gene silencing and antibody-mediated inhibition of Wnt-11 in colorectal cancer cell lines expressing high Wnt-11 reduced their capacity for invasion. Together, these observations suggest that Wnt-11 could be a potential target for the treatment of patients with invasive colorectal cancer.

## 1. Introduction

A hallmark of cancer is the re-activation of signals important during embryonic development. Among these, signals mediated by Wnt-11, which control cell movement in the embryo, are implicated in breast, colorectal, and prostate cancer cell migration and invasion (reviewed in [1]). WNT11 expression can be induced by many factors, including TGF-β, retinoic acid and β-catenin [1], and by environmental changes, such as hypoxia [2] and bacterial infection [3]. In the mouse intestinal epithelium, Wnt11 expression is associated with differential epithelial cell proliferation and migration in the proximal colon (high Wnt11), as compared to the distal colon (low Wnt11), and is reduced in mice lacking commensal bacteria, and this is reversed by conventionalization of germ-free mice [4]. In addition, a recent dietary intervention in humans found a significant negative correlation between WNT11 expression and fruit and vegetable intake [5]. WNT11 mRNA expression has been reported to be upregulated in an analysis of 133 primary colorectal tumors and 41 non-tumor tissues from a Japanese cohort, correlating with recurrence after surgery [6]. In colorectal cancer (CRC), mutations in APC or, in a minority of a cases, CTNNB1, result in constitutive activation of β-catenin-dependent signals and high expression of β-catenin/TCF/LEF target genes. β-catenin-dependent signals signaling increase the expression of WNT11, but this is highly dependent on transcription factors in addition to TCF/LEF family members (reviewed in [1]), and it remains unclear if WNT11 is a direct β-catenin/TCF/LEF target gene in CRC.

Stable over-expression of WNT11 stimulates IEC6 intestinal epithelial cell proliferation, migration, and contact-independent growth [7] and increases HCT116 cell proliferation, migration, and invasion [6], and WNT11 silencing reduces HCT116 cell migration [8]. In addition, the secreted protein AGR-2 (Anterior gradient protein 2 homolog) increases CRC cell migration by increasing WNT11 expression [9]. Wnt-11 activates intracellular kinases and transcription factors of the AP-1 (activator protein-1) and ATF/CREB (activating transcription factor/cAMP response element binding protein) families to regulate cell motility, cell–cell interactions and tissue morphogenesis. WNT11 mRNA expression is correlated with that of FZD7, raising the possibility that FZD_7_ transduces Wnt-11 signals in CRC. Consistent with this, FZD7 gene silencing reduces migration of WNT11-transfected HCT116 cells [6]. However, FZD7 gene silencing also reduces Wnt-β-catenin signaling [10] and FZD_7_ is also a receptor for Wnt-3A [11,12], which increases CRC cell proliferation [13], and for Wnt-2B, which drives the mesenchymal to epithelial transition (MET) [14]. Moreover, FZD_8_ transduces Wnt-11 signals that promote prostate cancer cell migration, invasion and the epithelial mesenchymal transition (EMT) [15]. Thus, further studies are needed to establish which FZD receptor(s) transduce Wnt-11 signals in CRC. Similarly, there are several candidate Wnt-11 co-receptors that are overexpressed in CRC, including PTK7 [16,17] and ROR1 [18].

The main aim of this study was to analyze Wnt-11 protein expression in CRC patient tumors. Wnt-11 levels were found to be increased in a subset of tumors and this was associated with patient gender, tumor location, tumor invasion, and patient mortality. We further found that Wnt-11 inhibits β-catenin/Tcf-dependent transcriptional activity and increases ATF2-dependent transcriptional activity and that inhibition of Wnt-11 reduces CRC cell invasion. Together, these observations highlight Wnt-11 as a potential biomarker and therapeutic target in CRC.

## 2. Results

### 2.1. Increased Expression of WNT11 and Potential Wnt-11 Receptors is Associated with Poor Prognosis in CRC

**Figure 1:**
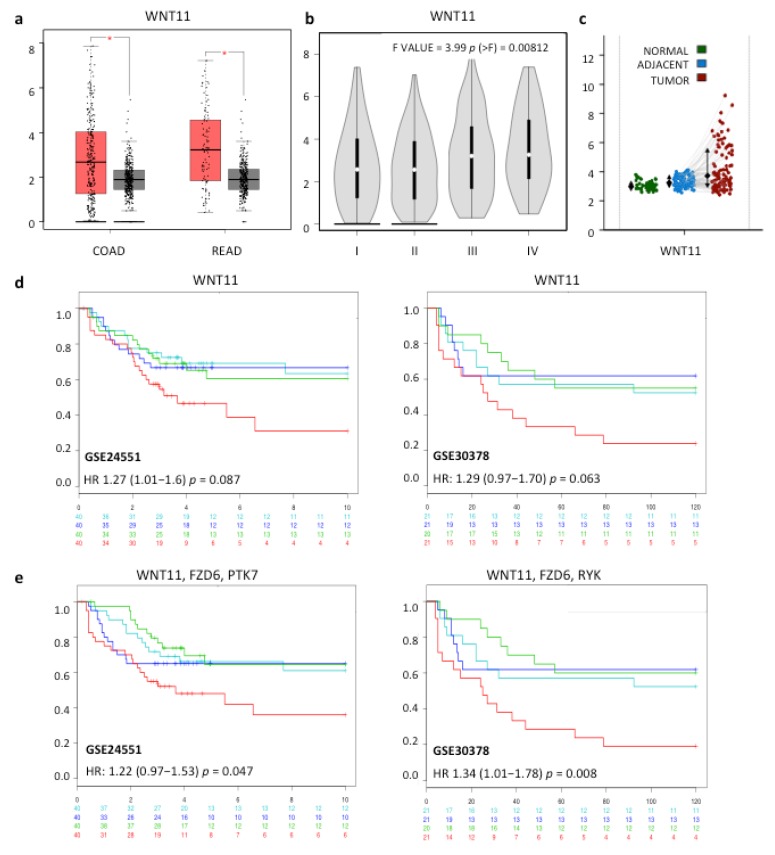
(**a**) Box plots comparing of WNT11 mRNA levels in normal/benign samples (from GTEx and TGCA, grey bars) and tumors (COAD and READ from TGCA, red bars) of the colon and rectum, respectively, analyzed using GEPIA, with fold-change set at 1.7 and *p* < 0.05; (**b**) Violin plots of WNT11 expression in the same COAD and READ TGCA datasets as in (**a**), according to stage of disease; (**c**) WNT11 expression in healthy colon mucosae (green, *n* = 50), normal adjacent mucosa (blue) and tumor(red) samples (*n* = 98) from the GSE44076 dataset, analyzed using Colonomics; increased WNT11 in tumors versus normal *p* = 8.9 × 10^−11^ (ANOVA and Tukey’s test); (**d**,**e**) Kaplan–Meier plots showing disease-free survival for two patient cohorts (GSE24551 [21], *n* = 160 and GSE30378 [22], *n* = 83) analyzed stratifying expression into equal groups (red (highest), dark blue, light blue, green (lowest) using SurvExpress [23]. Log–Rank *p*-values and numbers of surviving patients over time (GSE24551 years, GSE30378 months) are indicated, HR, hazard ratio (95% confidence intervals) estimated by fitting a Cox PR using risk group as covariate.

In order to confirm and extend the observations made in an earlier study [6], WNT11 mRNA gene expression levels were analyzed in public datasets using Oncomine (www.oncomine.org). WNT11 expression was significantly higher in CRC than in normal tissue in 7/11 datasets, with an overall increase in expression of 1.46-fold among 11 datasets comprising 1491 patients (*p* < 10^−8^) (Table 1 and Figure S1a). Further analysis using the GEPIA web tool (http://gepia.cancer-pku.cn/ [19]) to incorporate GTEx expression data from normal tissues confirmed elevated WNT11 mRNA expression in the TGCA CRC cohort (Figure 1a), with higher levels at Stages III and IV disease, compared to Stages I and II (Figure 1b). WNT11 expression was also elevated in tumors in a comparison of paired tumor samples and normal adjacent mucosa from a cohort of 98 patients and 50 healthy colon mucosae (GSE44076 [20]) using the Colonomics web tool (colonomics.org) (Figure 1c).

To identify potential Wnt-11 receptors in colorectal cancer, we examined Wnt receptor expression in the GSE44076 cohort. We found increased expression of FZD6, FZD7, RYK, and PTK7 in tumors, as well as a highly significant increase in FZD7 in tumor-adjacent cells (Table 2 and Figure S1b). This was confirmed in the TGCA CRC dataset using Oncomine (Table 2). Further analysis of the TGCA CRC dataset using GEPIA to incorporate expression levels in normal tissue confirmed the increases in FZD6 and PTK7. In contrast, there was no change in RYK and there was a significant reduction in FZD7 (Figure S1c). In addition, analysis using GEPIA found reduced mRNA expression of FZD4, FZD8, ROR1, and ROR2 and increased expression of LRP5 in the TGCA cohort (Table S1a). Correlation analysis found WNT11 expression was correlated with FZD6, RYK, and PTK7 expression in tumors and with FZD7 expression both in normal mucosae and tumors (Table 2 and Figure S2). Correlations were also observed in the TGCA dataset, analyzed using GEPIA (Table 2).

Given the increased expression of WNT11 in CRC and its correlation with FZD6/7, RYK, and PTK7, we determined if the expression of any of these genes might be informative with respect to patient prognosis. We noted trends in the TGCA dataset associating increased WNT11, FZD6, and PTK7 with lower disease-free survival (Figure S3). Further analysis of patient survival data from two patient cohorts (GSE24551 [21] and GSE30378 [22]) found trends for association of high WNT11 expression with reduced patient survival (Figure 1d and Figure S4). These associations were strengthened by analysis of combined expression of WNT11 with FZD6 and RYK and/or PTK7, but not with FZD7 (Figure S4). Importantly, stratification for high expression of WNT11, FZD6, and RYK (GSE24551) or PTK7 (GSE30378) found significant associations with reduced patient survival (Figure 1e). Together, these results confirm that WNT11 mRNA is increased in CRC, identify associations of WNT11 expression and the expression of three potential Wnt-11 receptors and provide further evidence that increased expression of WNT11, in combination with FZD6, RYK, and/or PTK7, could be an indicator of poor prognosis.

### 2.2. Increased Wnt-11 Protein Expression Associates with Poor Prognosis in Colorectal Cancer.

**Figure 2:**
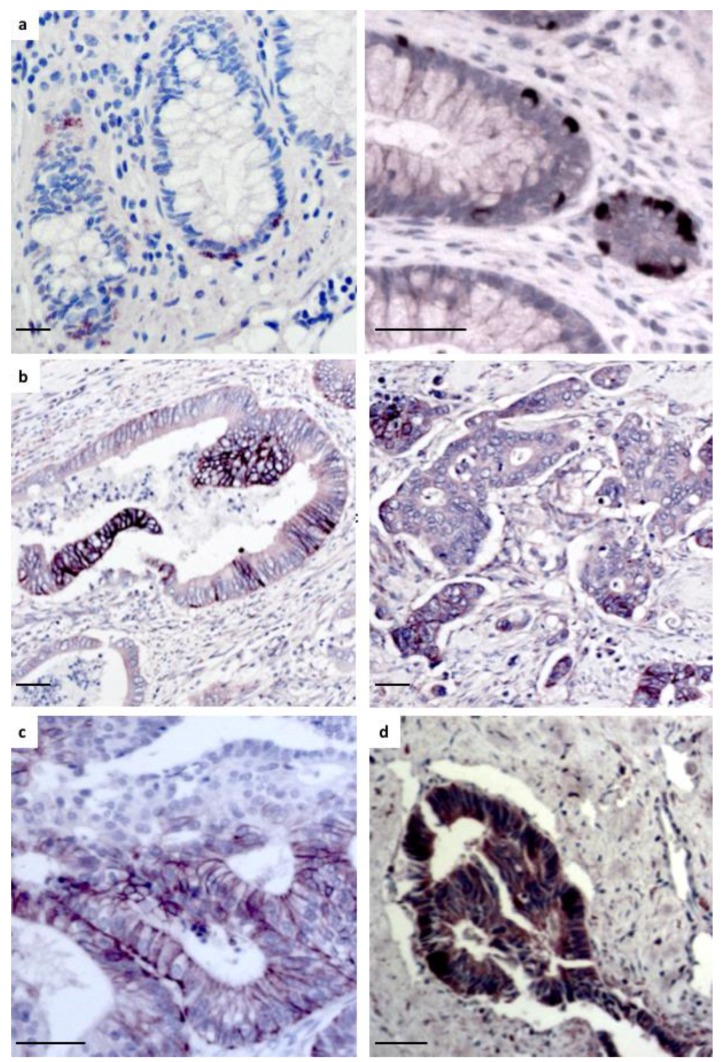
Immunohistochemical staining of Wnt-11. (**a**) Left: example of area of normal colon showing strongly Wnt-11-positive cells in red; right: higher magnification image highlighting strong Wnt-11 staining of cells close to base of crypts in brown; (**b**) left: example of a tumor with heterogeneous Wnt-11 staining, including strong signal at cell membranes/junctions; right: example of tumor with moderate Wnt-11 staining that is mostly intracellular; (**c**) example of tumor stained with a second anti-Wnt-11 antibody showing strong signal at cell membranes/junctions; and (**d**) example of a CRC liver metastasis with strong Wnt-11 staining in brown; scale bars 50 μm.

In order to determine the pattern of expression of Wnt-11 protein in CRC, we used antibodies previously optimized for detection of Wnt-11 in prostate cancer [15,24] to carry out immunostaining for Wnt-11 in benign and tumor areas of tumor sections from CRC patients. Wnt-11 was expressed at a low level in areas of normal colon, apart from high expression in a small number of cells that appeared to reside at the base of crypts (Figure 2a). Wnt-11 expression in tumors was heterogeneous, with some tumor cells showing intracellular staining and others showing very strong staining at cell–cell contacts (Figure 2b). Similar results were obtained using a second anti-Wnt-11 antibody (Figure 2c). In addition, Wnt-11 was found to be highly expressed in tumor cells from a CRC liver metastasis (Figure 2d).

To determine if Wnt-11 protein levels in CRC can also provide diagnostic or prognostic information, immunohistochemistry was used to detect Wnt-11 in samples from 357 CRC patients from hospitals in the Basque country. Scoring was carried out by two people (IG, RK), and confirmed by a pathologist (IZ). Staining for Wnt-11 in epithelial and tumor cells was classified as negative (−), low (±), moderate (+), high (++), or very high (+++); see Figure S5 for examples of each category. Wnt-11 levels were classified as very high (+++) in 43 (12%) and high in 86 (24%) of patients (Table 3).

**Figure 3:**
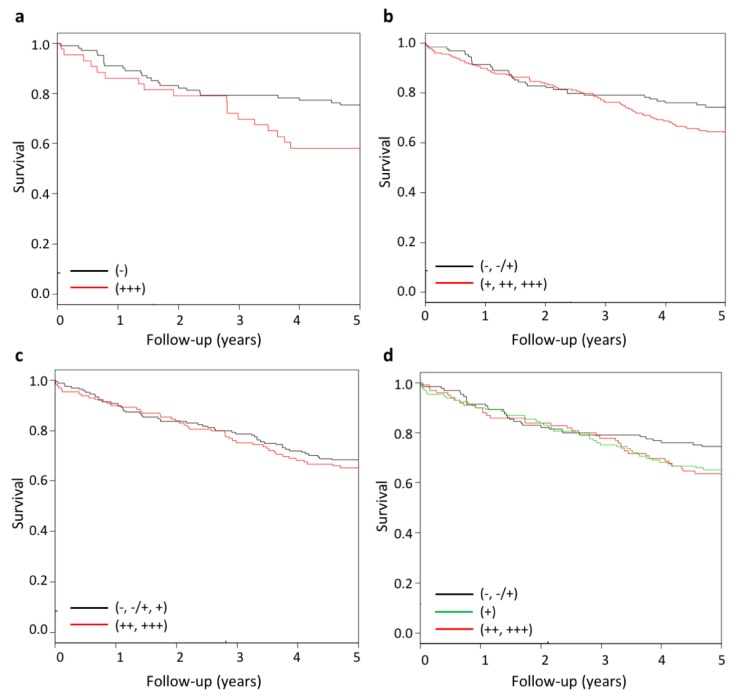
Kaplan–Meier curves at 5 years showing an increased risk of mortality rate in CRC patients with very high Wnt-11 levels. Patients were stratified for Wnt-11 levels as follows: (**a**) negative (−) and very high (+++), *p* = 0.04, (**b**) negative or low (−, ±) and moderate, high or very high (+, ++, +++), *p* = 0.08, (**c**) negative or low or moderate (−, ±, +) and high or very high (++, +++), *p* = 0.21, and (**d**) negative or low (−, ±), moderate (+) and high or very high (++, +++); *p* = 0.11 (+) and 0.13 (++, +++); Cox regression analysis, see Tables S5–S7 for HR, CI and unadjusted and adjusted analyses, and Figure S6 and Table S4 for 3-year mortality.

Analysis of Wnt-11 levels with respect to patient and tumor characteristics resulted in a number of interesting observations (Table 3, Tables S2 and S3). As expected, there was a significant association of Wnt-11 with the presence of adenocarcinoma (*p* = 0.006), and this was also observed for mucinous adenocarcinoma (*p* = 0.025). In addition, elevated Wnt-11 was more prevalent in rectal than in colon cancer (*p* = 0.04). There was a trend for higher Wnt-11 expression in tumors from women than from men (*p* = 0.07) that was significant when comparing patients with high or very high Wnt-11 and patients with moderate, low or no Wnt-11 (*p* = 0.01, Tables S2 and S3). The same comparison associated high Wnt-11 with tumor invasion (*p* = 0.04, Tables S2 and S3). There was no association between the level of Wnt-11 and KRAS gene mutation status or patient age. There was, however, an inverse relationship between Wnt-11 and the Charlson Comorbidity Index (*p* = 0.04). Analysis of patient survival data found an association between Wnt-11 and increased mortality at 5 years when comparing patients with very high Wnt-11 (+++) and patients negative for Wnt-11 (−) (Figure 3a, Tables S5–S7). There was also a trend for an association when comparing patients with very high, high, or moderate Wnt-11 and patients with no or low Wnt-11 (Figure 3b and Table S8). There was no trend when comparing patients with no, low or moderate Wnt-11 and those with high or very high Wnt-11 (Figure 3c and Table S7), or after trichotomization of patients into negative/low, moderate, and high/very high Wnt-11 (Figure 3d, Tables S9 and S10). Taken together, these results are reminiscent of those for WNT11 mRNA expression, which showed strong trends for an association with patient survival (Figure 1 and Figure S3a).

**Figure 4:**
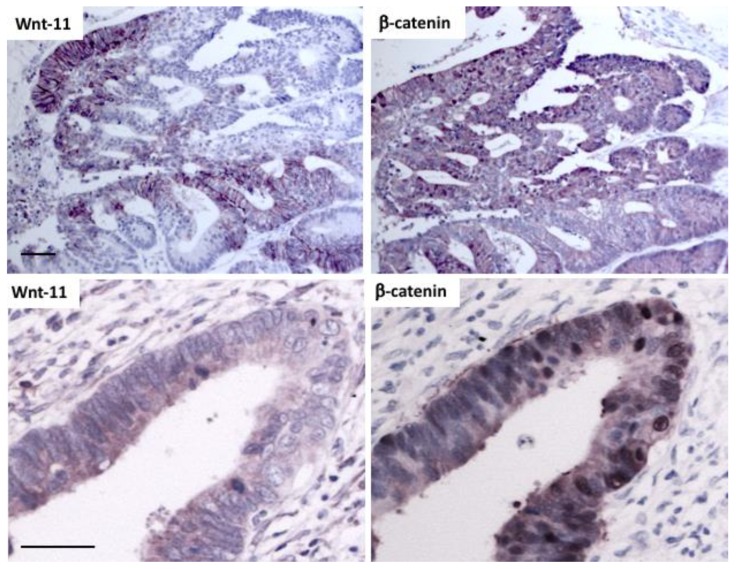
Comparison of Wnt-11 and β-catenin in CRC **Top**: immunohistochemical staining for Wnt-11 and β-catenin in adjacent sections of a CRC tumor (representative of samples from 5 patient tumors); note the lack of concordance of areas of cells with high Wnt-11 and high β-catenin. **Bottom**: higher magnification images of staining for Wnt-11 and β-catenin in adjacent sections of another CRC; scale bars 50 μm.

WNT11 is a β-catenin target gene in some contexts [1]. In order to determine if the elevated expression of Wnt-11 reflected activation of β-catenin, as indicated by its nuclear staining, adjacent sections of five tumors were stained for Wnt-11 and β-catenin. Like Wnt-11, β-catenin expression was also heterogeneous (Figure 4). Although there were areas where tumor cells expressed high levels of both Wnt-11 and β-catenin, other areas did not (Figure 4). The lack of overlap between Wnt-11 and β-catenin expression suggests that elevated Wnt-11 is not a direct result of β-catenin activation. However, a larger number of tumors will need to be examined to demonstrate this unequivocally. Nevertheless, the results in these patients are consistent with elevated Wnt-11 being a result of activation of signals in addition to or other than those regulated by β-catenin.

### 2.3. Inhibition of Wnt-11 Reduces Colorectal Cancer Cell Invasion

**Figure 5:**
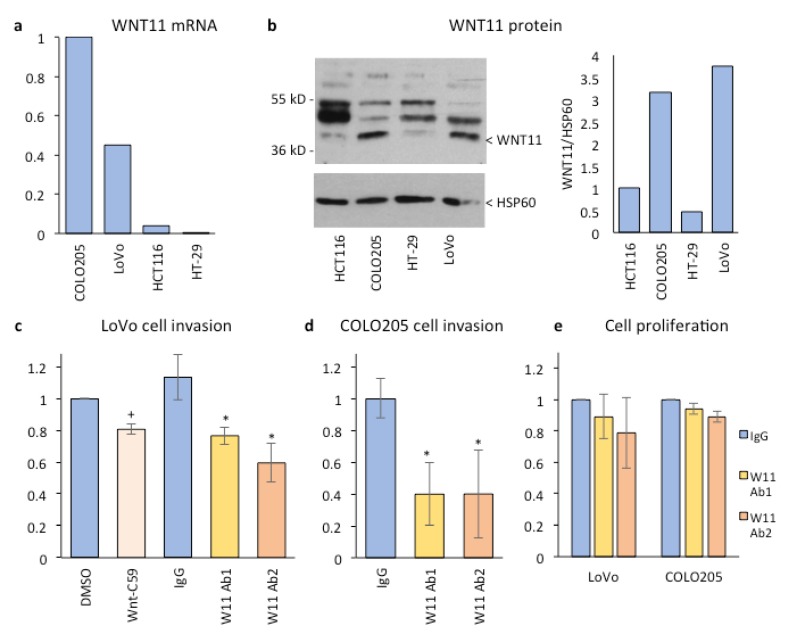

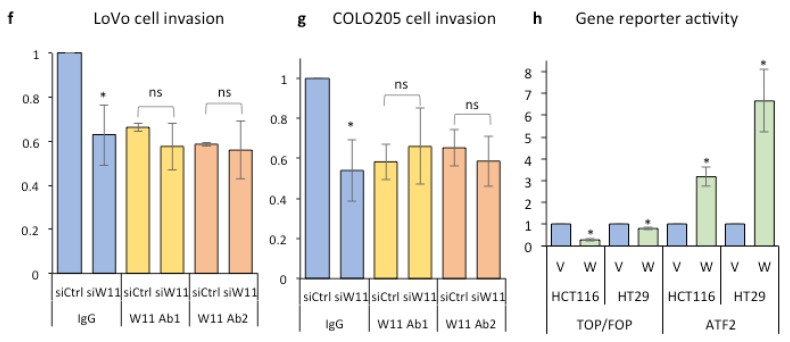
Wnt-11 expression and activity in CRC cell lines (**a**) q-RT-PCR for WNT11 mRNA in the indicated cell lines, showing average relative expression, normalized to COLO 205 cells; (**b**) cell extracts were probed with goat anti-Wnt-11 antibody and anti-HSP60 as loading control; arrowhead indicates position of Wnt-11, graph shows densitometry analysis; (**c**,**d**) relative invasion of LoVo cells (**c**) or COLO 205 cells (**d**) treated 24 h with 100 nM Wnt-C59 or vehicle (DMSO) (**c**) or 2 ug/mL rat anti-Wnt-11 (W11 Ab1/2) or rat IgG (**c**,**d**), *n* = 3, **^+^** *p* < 0.05 versus DMSO (Student’s *t*-test), * *p* < 0.05 versus IgG (ANOVA and Student’s *t*-test); (**e**) cell number normalized to control, after treatment with 2 ug/mL rat IgG or anti-Wnt-11 (W11 Ab1/2) for 5 days; (**f**,**g**) relative invasion of LoVo cells (**f**) and COLO 205 cells (**g**) transfected with control siRNA (siCtrl) or WNT11 siRNA (siW11) before plating for invasion and antibody treatment, *n* = 3, * *p* < 0.05 versus IgG siCtrl, ns, not significant; (**h**) relative activity of the indicated gene reporters in cells transfected with empty vector (V) or Wnt-11 plasmid (W) for 24 h, *n* = 3, * *p* < 0.05 versus empty vector (Student’s *t*-test).

To determine if Wnt-11 might be a potential target for therapy, we compared its expression in CRC cell lines to identify those that express high levels of Wnt-11. Quantitative RT-PCR analysis indicated that WNT11 mRNA levels were very high in COLO 205 cells (Ct WNT11–Ct 36B4 (ΔCt) ~6) and LoVo cells (ΔCt ~8), moderate in HCT116 cells (ΔCt ~12), and low in HT29 cells (ΔCt ~15) (Figure 5a), consistent with a previous study [6]. The Wnt receptors that correlated with WNT11 in patient tumors were highly expressed in all four cell lines, with FZD6 being the most abundant in LoVo cells (Figure S8a). We also examined the expression of WNT5A and WNT5B, as they are also non-canonical Wnts with altered expression in CRC (Table S1b). WNT5A was low/undetectable in all cell lines and WNT5B was high in LoVo cells and moderate/low in the other cell lines (Figure S8b), consistent with previous studies [25,26]. Western blots were carried out to confirm the WNT11 mRNA results at the protein level. As for WNT11 mRNA, Wnt-11 protein levels in cell extracts were higher in COLO 205 and LoVo cells than in HCT116 and HT-29 cells (Figure 5b); similar results were observed using cell conditioned media [27].

In order to determine if Wnt secretion is required for CRC cell invasion, invasion assays were carried out using LoVo cells treated with the porcupine inhibitor Wnt-C59, or carrier (DMSO) as a control. The results indicated that Wnt-C59 reduced LoVo cell invasion by 20% (Figure 5c), with similar results observed in COLO 205 cells (Figure S9), suggesting that Wnt secretion is required, to some extent, for CRC cell invasion. CRC cells express several Wnts and there are studies that indicate porcupine inhibition does not block secretion of all Wnt family members in all cell types [28,29]. We therefore used Wnt-11-specific antibodies (Figure S10a) and siRNA-mediated gene silencing (Figure S10b) to determine if endogenous Wnt-11 is required for CRC invasion. Wnt-11 antibodies inhibited LoVo cell invasion by 35–40% (Figure 5c) and COLO 205 cell invasion by 40–50% (Figure 5d). The invasion assay results were normalized to cell number, which was not significantly affected at this time point ([27] insert ref as above) or after 5 days of treatment (Figure 5e). In support of these results, WNT11 gene silencing reduced invasion of LoVo cells (Figure 5f) and COLO 205 cells (Figure 5g) to a similar extent as the Wnt-11 antibodies. CRC cell invasion was not further reduced by combined WNT11 gene silencing and Wnt-11 antibody treatment (Figure 5f,g), consistent with the antibodies being specific for Wnt-11. In prostate cancer cells, gene reporter assays have been used to show that Wnt-11 increases non-canonical Wnt signaling [15], measured using an ATF2-dependent luciferase reporter [30], and inhibits canonical Wnt (β-catenin/Tcf-dependent) signaling [31]. We wished to determine how Wnt-11 affects these reporters in CRC cells. β-catenin/Tcf-dependent signaling can be measured as the ratio of the luciferase reporters Super8xTOPFlash, which contains Tcf/LEF binding sites and Super8xFOPFlash, which has mutations in these sites. CRC cells have higher basal β-catenin/Tcf activity than prostate cancer cells, where the TOP/FOP ratio is below 1, while it is around 10 in HCT116 cells and 2–3 in HT29 cells ([27] insert ref as above). Wnt-11 inhibited β-catenin/Tcf-dependent activity both in HCT116 cells (TOP/FOP ratio 10.2 reduced to 2.6) and, to a lesser extent, in HT29 cells (TOP/FOP ratio of 2.6 reduced to 1.6) (Figure 5h). In addition, Wnt-11 increased ATF2-dependent gene reporter activity in both cell lines (Figure 5h), a similar extent as has been observed in prostate cancer cell lines [15]. In summary, Wnt-11 has similar effects on canonical and non-canonical Wnt signals, as measured by gene reporter assay, in CRC cells as in prostate cancer cells.

## 3. Discussion

The increased expression of WNT11 mRNA and Wnt-11 protein in CRC highlights the potential of this Wnt family member as a prognostic marker and therapeutic target in a subset of patients. Among our findings was that Wnt-11 protein levels were higher in tumors from females than from males. This might reflect hormone regulation of WNT11 expression, as WNT11 is downregulated by dihydrotestosterone in prostate cancer [31] and upregulated by estrogen in breast cancer [32]. However, we did not observe sex-specific differences in WNT11 mRNA levels in CRC in gene expression databases, so the difference could be particular to this patient cohort or reflect differences in protein rather than mRNA levels. Wnt-11 was also higher in rectal tumors than in colonic tumors, contrasting with the normal colon in mice, where Wnt11 gene expression is higher in the proximal colon than in the distal colon [4]. Although we did not observe significant location-specific differences in WNT11 expression in CRC datasets, analysis of TGCA and GTEx data using a higher cut-off found a more robust increase in WNT11 in rectal tumors than in colonic tumors (R.K., unpublished observations). A difference in rectal and colonic tumors could reflect differential regulation of Wnt-11 by the tumor environment, for example, WNT11 gene expression can be induced by hypoxia [2] and by bacterial infection [3], which may differ in the colon and rectum. Alternatively, it may reflect the cell type of tumor origin. Notably, there are colon and rectal-specific changes in the expression of several other genes in CRC [33].

There was a significant association of Wnt-11 expression and patient mortality at 5 years, but only when considering patient tumors with very high Wnt-11 (12% of the patients in this cohort). We also noted an inverse relationship between Wnt-11 and the Charlson Comorbidity Index, a factor for poor prognosis in rectal cancer patients in Spain [34] and in this patient cohort (Table S5). Thus, it appears that patients with high Wnt-11 have fewer co-morbidities, which may confound an association of Wnt-11 with increased mortality resulting from colorectal cancer. When patients were separated into Wnt-11 negative (−, ±) and Wnt-11-positive (+, ++, +++) groups, there was a trend for association with increased mortality in the Wnt-11-positive group, though not statistically significant. However, this was less apparent when trichotomizing into negative/low, moderate and high/very high groups. It is possible that there are too few patients for this type of analysis. Nevertheless, it is interesting to note that WNT11 mRNA expression also showed a trend for association with increased mortality (Figure 1d), which was significant only when combining expression with FZD6, RYK and/or PTK7 (Figure 1e).

The expression of Wnt-11 in CRC tumors only partially overlapped with that of nuclear β-catenin, suggesting that activation of β-catenin signaling *per se* does not account for the increase in Wnt-11 in patient tumors. This is consistent with ChIP analysis of β-catenin-Tcf/LEF target genes in the LS174t CRC cell line, where there was only weak regulation of WNT11 [35], and with WNT11 expression levels in CRC cell lines, which do not correlate with β-catenin/Tcf gene reporter activity. Studies in other cell types show that β-catenin induction of WNT11 mRNA expression depends on transcription factors in addition to Tcf/LEF family members [1], suggesting that increased Wnt-11 expression in patient tumors can provide information beyond the APC/CTNNB1 mutational status. As WNT11 expression can be induced by TGF-β [36] and by hypoxia [2], increased levels of Wnt-11 in patient tumors could be a result of activation of transcription factors that act independently or in association with β-catenin, such as Smad3 [37] and hypoxia inducible factor (HIF)-1α [38].

We noted different staining patterns of Wnt-11 in tumors, detecting it at cell membranes/junctions in some areas, suggesting it is secreted and accumulates at these sites, and mostly intracellular in other areas (Figure 1b). During embryonic development, Wnt-11 regulates cell–cell cohesion and cell contact persistence, affecting cell movement and tissue formation [39,40]. The localization of Wnt-11 to cell membranes/junctions in tumors may reflect its participation in related processes at these sites. Further studies are required to identify the receptors that transduce Wnt-11 signals in CRC. However, FZD_6_ is a strong candidate, being one of three FZDs that strongly colocalize with Wnt-11 in transfected cells (the others are FZD_8_ and FZD_10_) [15]. In addition, FZD6 mRNA levels are upregulated in CRC [41,42] and correlate with WNT11 mRNA expression and poor prognosis. RYK and PTK7 are candidate Wnt co-receptors, as their expression levels correlate with that of WNT11, and PTK7 is implicated in CRC [16,17].

In our cell line studies, WNT11 was found to be more highly expressed in COLO 205 and LoVo cells. The higher WNT11 expression in these cell lines, compared to in HCT116 and HT29 cells, does not seem to be related to their mutation status, as in this respect COLO 205 cells (mutant APC, CTNNB1, P53, and BRAF) are more similar HT29 cells (mutant APC, P53 and BRAF) and LoVo cells and HCT116 cells both have a KRAS mutations. Notably, WNT11 expression in HT29 cells, although low among CRC cell lines, was similar to what we observe in prostate cancer cell lines, where it promotes invasion [15], suggesting that Wnt-11 in HT29 cells may be functional. A more relevant difference among the CRC cell lines examined, perhaps, is that COLO 205 and LoVo cells are derived from metastases, whereas HCT116 and HT29 cells are from primary tumors, suggesting Wnt-11 plays a role in metastatic cancer. Consistent with this, siRNA-mediated gene silencing of WNT11 and antibody inhibition of Wnt-11 reduced LoVo and COLO 205 cell invasion. These results suggest that Wnt-11 is a major Wnt family member promoting invasion in these cell lines, despite LoVo cells expressing high levels of WNT5B, which increases CRC cell invasion, at least when overexpressed [26]. It should be noted, however, that WNT5B mRNA is downregulated in patient tumors (Figure S2a). In contrast to WNT5B, WNT5A is upregulated in patient tumors, as is WNT2, which increases β-catenin signaling and CRC cell proliferation [43]. High WNT5A expression has been associated with poor prognosis in another patient dataset [44], although the authors proposed that this may relate to expression by macrophages [45]. Our analysis of gene expression data using GEPIA found no association between WNT2 or WNT5A gene expression and patient survival. Indeed, ectopic WNT5A expression does not promote tumor progression [44,46]. Nevertheless, targeting Wnt-11 may require patient stratification to identify those patients with tumors expressing high levels of Wnt-11. In addition, some patients may benefit from combined targeting of Wnt-11, Wnt-5A, and/or Wnt-2, for which function-blocking antibodies have been described [43,47].

## 4. Materials and Methods

### 4.1. Cell Culture and Reagents

Cell lines were obtained from the American Type Culture Collection (HCT116 cells), Spiros Linardopoulos, Institute of Cancer Research, London (COLO 205 cells) and Laki Buluwela, Imperial College London (HT29 and LoVo cells). HCT116 and HT29 cells were cultured in McCoy’s 5A medium supplemented with 10% fetal bovine serum (FBS; Thermo Fisher Scientific, Madrid, Spain) and antibiotics (100 U/mL penicillin, 100 µg/mL streptomycin (Life Technologies S.A., Madrid, Spain). LoVo cells and COLO 205 cells, the latter grow loosely attached and in suspension, were cultured in DMEM and RPMI-1640, respectively, with GlutaMAX™ (Life Technologies), 10% FBS and antibiotics. All cells were cultured at 37 °C in 5% CO_2_, passaged for up to 6 months before replacement from early-passage frozen stocks and were regularly screened for mycoplasma.

### 4.2. RNA Extraction, cDNA Synthesis, and Quantitative Real Time PCR

RNA from cell lines was extracted using illustra™ RNAspin Mini Isolation Kit (GE Healthcare, Bilbao, Spain) according to manufacturer’s instructions). Cells were washed twice with PBS and then 350 μL of RA1 lysis buffer with β-mercaptoethanol added. RNA purity and quantity were measured using a Nanodrop spectrophotometer and 2 μg of total RNA was transcribed using M-MLV Reverse Transcriptase and RNaseOUT (Life Technologies). Quantitative PCR was performed using PerfeCTa SYBR^®^ Green Supermix, Low Rox (Quanta, Barcelona, Spain) in a Viia7 Real-Time PCR System (Applied Biosystems, Madrid, Spain) with the following conditions: Taq polymerase activation 95 °C 3 min, denaturation 95 °C 15 s, annealing/extension 62 °C 1 min, melting curve 95 °C 15 s, 60 °C 1 min, 95°C 15 s, 40 cycles. The ∆∆Ct quantitation method was used to determine mRNA fold changes in gene expression, with 36B4 as the housekeeping gene. Sequences of primers are published [15,48,49].

### 4.3. Protein Extraction and Western Blotting

Cells were plated in 6-well plates in 2 mL complete medium and, after 24 h, changed to 1 mL OPTIMEM (Life Technologies) for 48 h. After this time, conditioned media (CM) were collected and cells were washed twice with PBS. Total cell extracts were obtained by lysis in Millipore RIPA Lysis Buffer (50 mM Tris-HCl, pH 7.4, 150 mM NaCl, 0.25% deoxycholic acid, 1% NP-40, 1 mM EDTA) with cOmplete™ EDTA-free Protease Inhibitor Cocktail (Sigma-Aldrich S.A., Madrid, Spain), PhosSTOP phosphatase inhibitors (Sigma-Aldrich) and 0.1% SDS (Life Technologies). CM were centrifuged to remove dead cells and transferred to cold tubes. CM proteins were concentrated by adding 10 μL StrataClean Resin (Agilent Technologies S.L., Madrid, Spain) and vortexing for 20 s. Tubes were then incubated on ice for 2 min, centrifuged 2000× *g* for 2 min, supernatants discarded and the resin pellets resuspended in 10 μL 2×Laemmli Sample Buffer (SB, 4% SDS, 20% glycerol, 10% 2-mercaptoethanol, 0.004% bromophenol blue and 125 mM Tris HCl, pH 6.8). Cell lysates were centrifuged 12 min at 15,000× *g* and supernatants transferred to fresh tubes on ice, mixed with an equal volume of SB. Samples were heated 3 min at 95 °C and separated on SDS polyacrylamide gels using a Mini Protean System (Bio-Rad Laboratories, S.A., Madrid, Spain) and transferred to nitrocellulose using a Trans–Blot^®^ SD Semi–Dry Electrophoretic Transfer Cell (Bio-Rad). Blots were rinsed in TBS-T (TBS, 0.05% Tween 20 (Life Technologies)), incubated in blocking buffer (TBS-T with 3% BSA (Sigma-Aldrich)) for at least 1 h and then incubated overnight at 4 °C with primary antibodies (goat anti-Wnt-11 (AF2647, Bio-Techne R&D Systems, S.L., Madrid, Spain), 1:2000), rabbit anti-HSP60 (sc-13966, 1:2000), mouse anti-Wnt-3a (sc-136163, 1:1000) (Santa Cruz Biotechnology, Heidelberg, Germany) and rat anti-Wnt-11 (Ab1, 3.5 μg/mL, GenScript USA Inc, Piscataway, NJ, USA). After washing with TBS-T, blots were incubated for 1 h in blocking buffer with HRP-conjugated secondary antibodies (Jackson ImmunoResearch Europe Ltd, Ely, UK) diluted 1:20,000. Membranes were developed using chemiluminescence (Amersham ECL Western Blotting Detection Reagents, GE Healthcare).

### 4.4. Clinical Samples

Tumor tissues were obtained from 358 colorectal cancer patients from Galdakao hospital (Galdakao, Spain) and Basurto hospital (Bilbao, Spain), following patient consent and approval from the local research ethics committee (see [50]). The clinical characteristics of the patients are summarized in Table 3. Staining of adjacent sections using control, Wnt-11 and β-catenin antibodies was carried out using samples from five of the patients.

### 4.5. Immunohistochemistry

Tissue sections were de-paraffinized with Histo-Clear II (National Diagnostics, Atlanta, GA, USA) and then transferred through four changes of 100%, 96%, and 70% ethanol and water. Antigen retrieval was performed in a pressure cooker filled with sodium citrate buffer at pH 6.0. Endogenous peroxidase activity was quenched for 10 min with 3% hydrogen peroxide. Blocking was performed for 15 min with Avidin followed by 15 min with Biotin (Avidin/Biotin blocking kit , Vector Labs, Burlingame, CA, USA). Samples were washed with PBS and blocked with 5% horse serum for 30 min at room temperature to reduce nonspecific staining. After washing, primary antibodies to Wnt-11 (Bio-Techne R&D AF2647 at 1:200 and Genetex GTX105971 at 1:50) and β-Catenin (BD610154 at 1:250) were applied overnight at 4 °C. Sections were incubated with biotinylated secondary antibody (Vector Labs) for 30 min followed by Vectastain^®^ Elite ABC reagent (Vector Labs) for 30 min. Liquid diaminobenzidine (DAB) (DAKO) was used as a chromogenic agent for 1–2 min and sections were counterstained with Mayer’s hematoxylin. Images were taken on an AxioImager D1 light microscope (Carl Zeiss Iberia SL, Madrid, Spain).

### 4.6. Invasion and Proliferation Assays

For invasion assays, 50,000 LoVo and COLO 205 cells per well in DMEM and RPMI, respectively, containing 1% FBS, were added to duplicate Matrigel-coated 8 µm pore Transwell filters with a polycarbonate membrane (Corning S.L.U., Madrid, Spain). Inserts were set in 24-well plates with media containing 20% FBS in the lower chamber for 24 h. Non-invading cells were removed using a cotton swab and invading cells were stained using 0.1% crystal violet, 20% methanol, and 0.36% paraformaldehyde (PFA) in PBS. Pictures were taken of 6 different fields using a 10× objective and the average numbers of invading cells per insert was determined by counting stained cells. Proliferation assays were carried out by plating 4000 cells per well in 24-well plates with media replaced on day 2. On day 5, cells were stained using crystal violet. After drying, crystal violet was solubilized in 10% acetic acid and absorbance measured at 595 nm using a Synergy HT plate reader (BioTek, Bad Friedrichshall, Germany). Wnt-C59 (Cellagen Technology, San Diego, CA, USA) was resuspended in DMSO, diluted in medium, and used at 100 nM. Rat anti-Wnt-11 and control rat IgG (Sigma-Aldrich) were used at 2 μg/mL. The rat anti-Wnt-11 antibodies Ab1 and Ab2, raised to Wnt-11 peptides, recognize recombinant Wnt-11 but not Wnt-3a (Figure S10) or Wnt-5a ([27] insert ref as above); a detailed characterization of these antibodies is in progress. For gene silencing, 2 × 10^5^ cells were plated in 6-well plates in complete media. The following day, cells were transfected with 50 nM control and WNT11 (L-009474-00-0005) ON-TARGETplus SMARTpool siRNAs using Lipofectamine RNAiMAX (Thermo Fisher Scientific, Spain), according to the manufacturer’s instructions. Fresh media were added to the cells 4 h after transfection. After 48 h, cells were trypsinized and resuspended in media containing 1% FBS. 50,000 cells were added to Transwell filters, with antibodies, as described above. The same numbers of cells were plated in parallel to normalize invasion assay results to cell number and to determine the extent of WNT11 gene silencing (Figure S10b).

### 4.7. Transient Transfections and Gene Reporter Assays

For gene reporter assays, 75,000 cells were plated in triplicate in 12-well plate. After 24 h, cells were washed with OptiMEM and transfected with ATF2-luc, Super8xTOPFlash or Super8xFOPFlash (250 ng), pRL-tk (50 ng) and either 200 ng empty vector or WNT11 expression plasmid using Lipofectamine LTX with PLUS (Life Technologies), as instructed by the manufacturer. Plasmids were previously described [15,49,51]. Fresh media were added to the cells 4–6 h after transfection. 24 h after transfection, cells were washed twice with PBS and lysed in Passive Lysis Buffer (Promega Biotech Ibérica, S.L., Madrid, Spain). Luciferase activity was measured using the Dual Glo Luciferase Assay System (Promega) or Luciferase Assay Kit (PJK GmbH, Kleinblittersdorf, Germany), as instructed by manufacturers. Gene reporter activities were calculated as ATF-2-luc/renilla and Super8xTOP/renilla/Super8xFOP/renilla ratios.

### 4.8. Bioinformatics Analysis of Gene Expression Data

Publicly available gene expression data were analyzed using software associated with the web tools indicated in the text and figure legends. SurvExpress (http:/bioinformatica.mty.itesm.mx:8080/Biomatec/SurvivaX.jsp) [23] and PROGgeneV2 (http://genomics.jefferson.edu/proggene/) [52] were used for Kaplan–Meier analysis and Cox proportional hazard regression, bifurcating at the median level of expression; Log−Rank Equal Curves *p*-values were determined using SurvExpress.

### 4.9. Statistical Analysis

For Wnt-11 staining data, an exploratory data analysis of the sample was performed, calculating means and standard deviations for continuous variables and frequencies and percentages for categorical ones. Chi-Square test (or Fisher’s Exact test, when needed) and the Wilcoxon and Kruskal–Wallis non-parametric tests were applied to assess the relationship between clinical variables and the different Wnt-11 staining classifications. The same statistical procedures were used to identify potential predictors of 3- and 5-year mortality. Finally, using the backward procedure, multivariate Cox regression models were developed for the prediction of 3- and 5-year mortality. To this end, those variables with a *p*-value < 0.20 in the previous step were considered as explanatory variables. Results are shown as Hazard ratios with their confidence intervals. Statistical procedures were all performed using SAS System v9.4 and the pictures depicted with R 3.5 release. All *p*-values were deemed to be statistically significant if *p* < 0.05. For gene reporter, invasion and proliferation assays, two-sided Student’s t-test for single comparisons or one-way analysis of variance (ANOVA) with post hoc Tukey´s test for multiple group comparisons were used. A two-tailed *p*-value ≤ 0.05 was considered to indicate statistical significance.

## 5. Conclusions

The observations made in this study highlight the potential of Wnt-11 as a possible target for the treatment of patients with invasive colorectal cancer. The results are of interest because they show location- and gender-specific differences in Wnt-11 protein levels in colorectal cancer and because they suggest that antibody-mediated inhibition of Wnt-11 should be considered as a possible approach for future therapies for metastatic CRC. The study also identifies FZD6, RYK, and PTK7 as candidate receptors for Wnt-11 in colorectal cancer.

## Figures and Tables

**Table 1:** WNT11 mRNA expression in CRC datasets ^1^.

Dataset	Patients	Fold Change	*p*-Value
Bittner Colon	373	1.513	0.058
Gaedke Colorectal	130	1.75	8.8 × 10^−9^
Gaspar Colon	78	1.187	0.021
Hong Colorectal	82	4.914	5.18 × 10^−11^
Jorissen Colorectal 3	154	1.468	0.19
Kaiser Colon	105	1.301	0.002
Ki Colon	123	−1.251	0.997
Sabates-Beliver Colon	64	1.3	0.168
Skrzypczak Colorectal	105	1.268	0.003
Skrzypczak Colorectal 2	40	1.404	0.000165
TGCA	237	1.713	1.2 × 10^−14^
All	1491	1.46	8.88 × 10^−98.9^

^1^ analyzed using Oncomine (see also Figure S1a).

**Table 2:** Summary of WNT11 and Wnt receptor expression analysis in CRC

**Gene**	***p* (Normal vs. Adjacent) ^1^**	***p* (Normal vs. Tumor)2**
WNT11	0.12	8.9 × 10^−11^, 1.21 × 10^−14^
FZD6	0.034	1 × 10^−12^, 2.32 × 10^−18^
FZD7	5.3 × 10^−12^	2.7 × 10^−9^, 2.24 × 10^−8^
RYK PTK7	0.0015 0.81	<2 × 10^−16^, 4.24 × 10^−10^ <2 × 10^−16^, 6.3 × 10^−18^
**Gene Pair**	**Correlation (Pearson)**	***p*-Value**
WNT11/FZD6	0.311, 0.17	0.00181, 0.00093
WNT11/FZD7	0.242, 0.11	0.0161, 0.028
WNT11/RYK WNT11/PTK7	0.239, 0.29 0.283, 0.27	0.0176, 0.000000024 0.00475, 0.00000017
**WNT11**	**Comparison**	***p*-Value**
LEFT vs. RIGHT	Tumor 4.51, 3.87	0.047
LEFT vs. RIGHT	Adjacent 3.42, 3.19	0.002
LEFT vs. RIGHT	Normal 3.17, 2.95	0.007
Gender	Males 4.28, Females 4.2	NS
k-RAS	Yes 4.3, No 4.15	NS
Age	Lower in older patients	0.0073

^1^ normal versus (vs.) adjacent comparison for GSE44076; ^2^ normal vs. tumor and correlation for GSE44076 (1st value) and TGCA (2nd value); NS, not significant.

**Table 3:** Patient sociodemographic and clinical features, according to Wnt-11 classification ^1^.

Wnt-11 *N* (%)	(−) 101 (28)	(±) 28 (8)	(+) 99 (28)	(++) 86 (24)	(+++) 43 (12)	Total 357	*p*
**Gender**							0.07
Male	65 (64.4)	19 (67.9)	76 (76.8)	49 (57)	27 (62.8)	236 (66.1)	
Female	36 (35.6)	9 (32.1)	23 (23.2)	37 (43)	16 (37.2)	121 (33.9)	
**Age, Mean (s.d.)**	69.9 (11.5)	69.4 (12.6)	70.5 (10.0)	69.1 (11.1)	71.7 (10.6)	70.0 (11)	0.79
**Age**							0.72
<49	5 (4.95)	2 (7.1)	3 (3)	6 (7)	0 (0)	16 (4.4)	
50–59	16 (15.8)	3 (10.7)	10 (10.1)	11 (12.8)	8 (18.6)	48 (13.4)	
60–69	19 (18.8)	6 (21.4)	21 (21.2)	22 (25.6)	8 (18.6)	76 (21.3)	
70–79	42 (41.6)	11 (39.3)	49 (49.5)	33 (38.4)	15 (34.9)	150 (42)	
≥80	19 (18.8)	6 (21.4)	16 (16.2)	14 (16.3)	12 (27.9)	67 (18.8)	
**CCI**							0.04
≤2	50 (49.5)	14 (50.0)	46 (46.5)	44 (51.2)	25 (58.1)	179 (50.1)	
3	23 (22.8)	3 (10.7)	36 (36.4)	17 (19.8)	11 (25.6)	90 (25.2)	
≥4	28 (27.7)	11 (39.3)	17 (17.2)	25 (29.1)	7 (16.3)	88 (24.7)	
**Adenocarcinoma**	88 (87.1)	22 (78.6)	95 (96)	83 (96.5)	40 (93.0)	328 (91.9)	0.006
**Mucinous Ad.**	9 (8.9)	6 (21.4)	4 (4)	4 (4.7)	3 (7)	26 (7.3)	0.025
**Tumor Location**							0.04
Rectal	36 (35.6)	7 (25)	16 (16.2)	21 (24.4)	10 (23.3)	90 (25.2)	
Colon	65 (64.4)	21 (75)	83 (83.8)	65 (75.6)	33 (76.7)	267 (74.8)	
**Tumor Invasion**							0.61
No invasion	90 (89.1)	25 (89.3)	83 (83.8)	75 (87.2)	39 (90.7)	312 (87.4)	
1 organ	10 (9.9)	3 (10.7)	16 (16.2)	9 (10.5)	3 (7)	41 (11.5)	
>1 organ	1 (1)	0 (0)	0 (0)	2 (2.3)	1 (2.3)	4 (1.1)	
**K-ras Mutation**							0.72
Not determined	95 (94)	27 (96)	92 (93)	79 (92)	41 (95)	334 (94)	
negative	2 (2)	0 (0)	5 (5)	3 (3.5)	0 (0)	10 (2.8)	
positive	4 (4)	1 (4)	2 (2)	4 (4.6)	2 (4.7)	13 (3.6)	
**Mortality**							
3 years	21 (20.8)	6 (21.4)	22 (22.2)	19 (22.1)	13 (30.2)	81 (22.7)	0.80
5 years	25 (24.8)	8 (28.6)	36 (36.4)	28 (32.6)	18 (41.9)	115 (32.2)	0.25

^1^ Wnt-11 staining was classified as negative/not detected (−), low (±), moderate (+), high (++) or very high (+++); CCI Charlson Comorbidity Index; numbers in parentheses are % unless indicated; s.d. standard deviation; Ad. Adenocarcinoma. Bold: *p* < 0.05.

## Supplementary Materials

The following are available online at https://www.mdpi.com/2072-6694/11/7/908/s1, Figure S1: Expression analysis of WNT11 and Wnt receptors in colorectal cancer, Figure S2: Correlation analysis of WNT11 and Wnt receptor expression in colorectal cancer, Figure S3: Survival analysis of WNT11 and Wnt receptor expression in CRC (TGCA dataset), Figure S4: Survival analysis of WNT11 and Wnt receptor expression in CRC (other datasets), Figure S5: Wnt-11 immunohistochemistry scoring examples, Figure S6. Kaplan–Meier curves stratifying CRC patients for Wnt-11 expression levels at 3 years, Figure S7. Kaplan–Meier curves stratifying CRC patients for Wnt-11 expression levels at 5 years, Figure S8: Expression of WNT and Wnt receptor genes in colorectal cancer cell lines, Figure S9: Effect of Wnt-C59 on COLO 205 cell invasion, Figure S10: Western blots of recombinant Wnt-11 and recombinant Wnt-3a and WNT11 gene silencing, Table S1: Analysis of WNT11 and Wnt receptor expression in colorectal cancer, Table S2: Comparison of patients with tumors having very high Wnt-11 versus moderate, low or no Wnt-11 (Figure 2a), Table S3: Comparison of patients with tumors having high or very high Wnt-11 versus moderate, low or no Wnt-11, Table S4: Unadjusted analysis for 3-year mortality, Table S5: Unadjusted analysis for 5-year mortality, Table S6: Adjusted analysis for 3- and 5-year mortality, Table S7: Summary of results of the prediction of 3- and 5-year mortality, Table S8: Summary of results of the prediction 5-year mortality (Figure 3b), Table S9: Patient sociodemographic and clinical features according to Wnt-11 classification in Figure 3d, Table S10: Summary of results of the prediction 5-year mortality (Figure 3d).
